# Health insurance and child mortality in rural Burkina Faso

**DOI:** 10.3402/gha.v8.27327

**Published:** 2015-04-28

**Authors:** Anja Schoeps, Henrike Lietz, Ali Sié, Germain Savadogo, Manuela De Allegri, Olaf Müller, Rainer Sauerborn, Heiko Becher, Aurélia Souares

**Affiliations:** 1 Institute of Public Health, University of Heidelberg, Heidelberg, Germany; 2 Centre de Recherche en Santé de Nouna, Nouna, Burkina Faso; 3 Institute for Medical Biometry and Epidemiology, University Medical Center Hamburg-Eppendorf, Hamburg, Germany

**Keywords:** health insurance, Africa South of the Sahara, child mortality, population surveillance, Burkina Faso, health services accessibility, survival analysis

## Abstract

**Background:**

Micro health insurance schemes have been implemented across developing countries as a means of facilitating access to modern medical care, with the ultimate aim of improving health. This effect, however, has not been explored sufficiently.

**Objective:**

We investigated the effect of enrolment into community-based health insurance on mortality in children under 5 years of age in a health and demographic surveillance system in Nouna, Burkina Faso.

**Design:**

We analysed the effect of health insurance enrolment on child mortality with a Cox regression model. We adjusted for variables that we found to be related to the enrolment in health insurance in a preceding analysis.

**Results:**

Based on the analysis of 33,500 children, the risk of mortality was 46% lower in children enrolled in health insurance as compared to the non-enrolled children (HR=0.54, 95% CI 0.43–0.68) after adjustment for possible confounders. We identified socioeconomic status, father's education, distance to the health facility, year of birth, and insurance status of the mother at time of birth as the major determinants of health insurance enrolment.

**Conclusions:**

The strong effect of health insurance enrolment on child mortality may be explained by increased utilisation of health services by enrolled children; however, other non-observed factors cannot be excluded. Because malaria is a main cause of death in the study area, early consultation of health services in case of infection could prevent many deaths. Concerning the magnitude of the effect, implementation of health insurance could be a major driving factor of reduction in child mortality in the developing world.

Reducing child mortality in developing countries has been one of the major foci of the United Nations Millennium Development Goals. The protection of the lives of young children will likely remain an important goal in the post-2015 agenda (1, 2).

Access to modern health services and medication is a key factor in the prevention of child deaths. Because individuals are charged user fees at the point of use, utilisation of health services is often linked with high and partly unexpected costs for the average household in disadvantaged populations. This expense often hampers access to modern health services, especially for young children (3). Health insurance schemes, such as community-based health insurance (CBHI), have been implemented in a number of countries to increase utilisation of health facilities and to avoid excessive out-of-pocket expenditure on health care, which can lead to poverty (4, 5). Such poverty can, in turn, lead to poor health and higher risk of death because it impedes the purchase of other health-related goods, such as food, clothing, or household items (6).

There is evidence that CBHI schemes have the ability to reduce out-of-pocket expenditure and increase utilisation of modern health services for people who are enrolled (6–8). The ultimate effect of such health insurance schemes on child mortality, however, has not yet been thoroughly investigated, mostly due to data unavailability in other settings (6, 7, 9).

## Objective

This study aims to investigate the effect of health insurance enrolment on mortality of children under 5 years of age in a health and demographic surveillance system (HDSS) in Nouna, Burkina Faso.

## Methods

### Study area

**Fig. 1:**
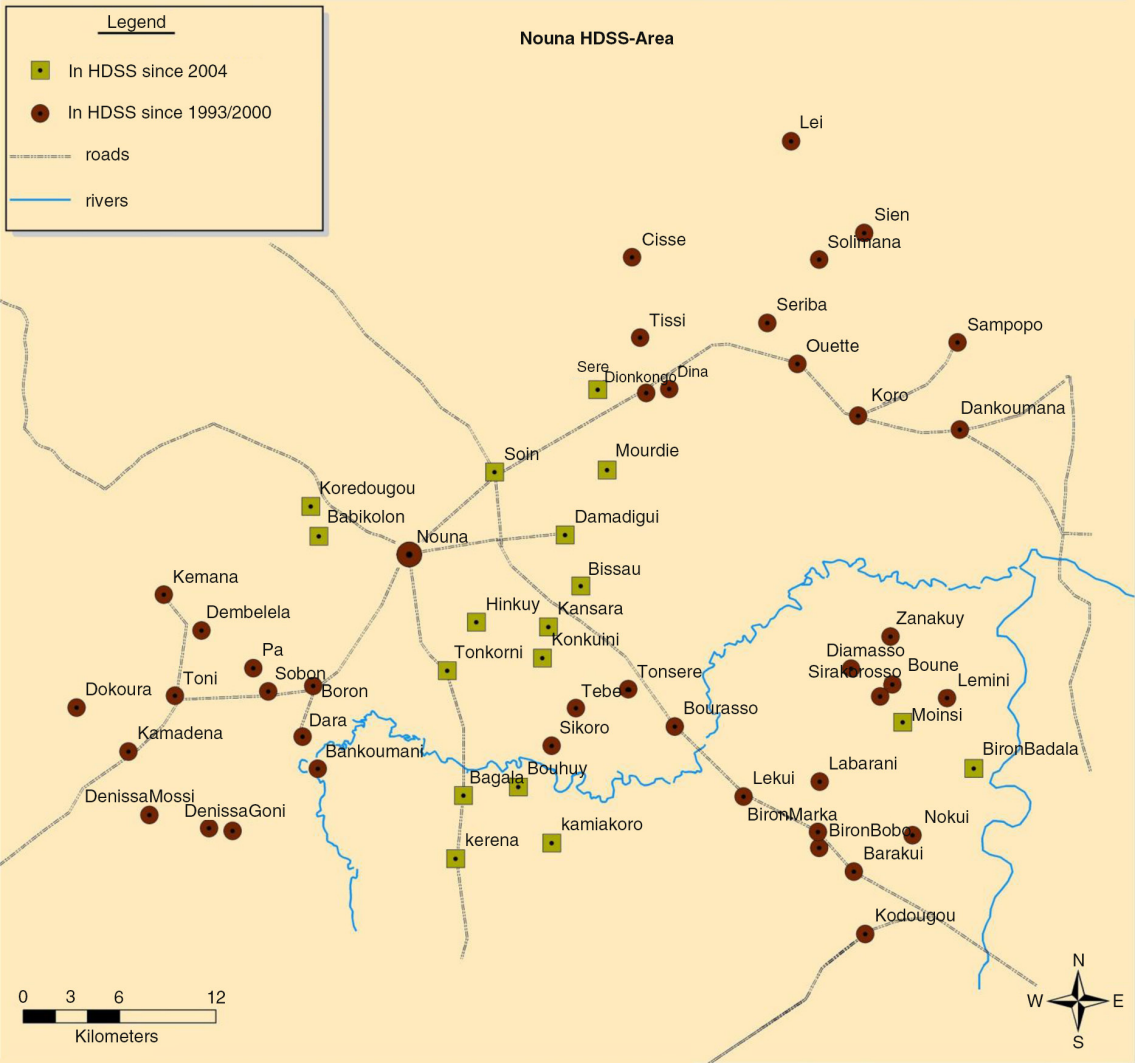
Detailed area map of the Nouna health and demographic surveillance system in Burkina Faso.

This study was conducted in the Nouna HDSS, which is located in a rural area of north-western Burkina Faso. In 2010, the Nouna HDSS area was inhabited by about 89,000 people of different ethnicities and religious beliefs, living in the semiurban town of Nouna and 58 surrounding villages (Fig. 1) (10). The Nouna HDSS was established by an initial census in 1992 and is a member of the INDEPTH network (10). Since then, all households in the study area have been visited three times a year to register vital events such as births, deaths as well as in- and out-migration, which are summarised in the routine HDSS database.

Health facility density in the Nouna HDSS has increased in recent years. Although the area was served by one district hospital in the town of Nouna and only three smaller health centres in 1992, the number of health centres had increased to 13 by the year 2009 (10). All facilities offer basic outpatient services, dispensaries, and maternity units.

### Community-based health insurance

A CBHI scheme was initiated in the Nouna HDSS area in 2004. It was offered to an increasing number of villages in three waves between 2004 and 2006. From 2006 onwards, insurance was offered to all residents of the early 41 villages of the rural Nouna HDSS and the population of the town of Nouna (Fig. 1) (11). The enrolment period is 1 year, after which insurance enrolment has to be renewed. The premium for adults is 1,500 CFA (US$3) yearly, whereas children under 15 years pay a reduced premium of 500 CFA (US$1) (12). Moreover, in the year 2007 a subsidy was introduced to cover 50% of the premium for households in the poorest quintile of the population. This effort was meant to enhance equity by encouraging enrolment among the most disadvantaged. The premium was set at an individual level to 750 CFA (US$1.50) for adults and to 250 CFA (US$0.50) for children (8, 13). Insured persons can access care free of charge at point of use, such as medication, laboratory tests, or x-rays at their reference health facility, which is generally the closest health facility. Health insurance also covers surgery and in-patient treatment for up to 15 days at the hospital in Nouna, but only if insured patients have been referred to the hospital from their local health facility. AIDS treatment, dental care, circumcision, or ophthalmology is not covered by the CBHI (12, 14).

### Data collection

Since the insurance rollout in 2004, information on all insured individuals in a respective year has been collected by the insurance administration, including date of subscription, birthdate, individual ID of the HDSS, and household ID. Using this information, data from the insurance can be linked with the database of the routine HDSS data collection. Due to the incompleteness of the individual ID numbers in the insurance files, about 89% of the insured persons could be linked with the HDSS routine database.

To get information about education and socioeconomic status (SES), data from a census conducted in 2009 were used. In addition to the baseline census, full censuses were conducted in the study area in 2000 and 2009 to check follow-up procedures of the HDSS and to gain additional information, such as asset ownership and housing characteristics. By use of the individual ID and household ID, census data could be linked with the routine HDSS database. Based on asset ownership (such as mobile phone, TV, or refrigerator), ownership of means of transport, agricultural material, and livestock, as well as housing characteristics (such as type of walls and roof, source of lighting, or the source of water), an index was constructed using principal component analysis (15). This index was constructed separately for rural and urban households, which were each divided into quintiles according to the SES score (16).

### Statistical analysis

CBHI was offered for the first time in 2004. This study included all individuals from the study area born in 2000 or after, so they had the chance to enrol at least once before their fifth birthday. The last date of observation was December 31st, 2010. Yearly under-five mortality rates were calculated for the group of enrolled and non-enrolled children separately. When a death was observed, it was attributed to the group of enrolled children if the child was enrolled at the time of death, and to the non-enrolled group otherwise. Likewise, the number of enrolled and non-enrolled person-years served as the denominators. To correct for different age-distributions among the enrolled and non-enrolled, rates for the enrolled were age-standardised by age-distribution of the non-enrolled group, stratified by the age-groups 0 months, 1–11 months, 12–23 months, 24–35 months, 36–47 months, and 48–59 months.

Two different Cox proportional hazards regression models were used for analysis. To avoid bias by data inaccuracy for very young deceased individuals, we excluded 708 children who died or were lost to follow-up in their first month of life. The remaining individuals entered the risk set on their 31st day of life. (However, a sensitivity analysis including all individuals, starting from their first day of life, yielded identical results.) The first model was used to identify determinants of health insurance enrolment and thereby enable adjustment for possible confounding in the main analysis, so the endpoint of the first model was enrolment into CBHI and age at first enrolment was used as the time variable.

We studied the following possible factors associated with enrolment in CBHI: sex, year of birth, rural vs. urban residence, distance to the reference health facility, ethnicity, religion, SES, household size, number of older siblings, twin vs. single birth, birth spacing to next younger sibling, age of mother at time of birth, age of father at time of birth, educational attainment of mother and father, mother surviving or deceased, and CBHI enrolment of the mother at the time of birth. Year of birth was used in the regression model as a continuous variable, taking values between 2000 and 2010. Household size, distance to the reference health facility, and previous death of the mother were included as time-dependent covariates. Household size was calculated separately at the middle of each calendar year, while distance to the health facility changed yearly in case of opening of a new health facility. If the mother died, the value of that variable was set to one from the day of the mother's death. All variables were tested in a crude model and in one full model containing all variables of interest. Upon showing an effect in either model, the variable was included in the final model presented.

The main regression model with the end point of under-five mortality included current insurance status as the main variable of interest and as adjustment factors the full set of variables from the final model with the end point of insurance enrolment. Age at death was the underlying time variable, so the remaining individuals were censored at their fifth birthday or at the time of loss to follow-up. Insurance enrolment status was included in the analysis as a time-dependent covariate, so multiple episodes of being insured and non-insured in a single individual could be taken into account. The value of insurance status for non-insured individuals was set to 0 and changed to 1 on the day of initial enrolment. When enrolment ended or was interrupted, the value was set back to 0. This value could change several times during the 5-year observation time of the individuals.

From a subsample of about one quarter of the study population, data on vaccination coverage with all recommended vaccinations at age 1 year was available (17). A sensitivity analysis was performed to study the effect of vaccination coverage on the association between insurance enrolment and mortality in children aged 1 to 4 years of age.

All analyses were performed using SAS 9.3.

### Ethics committee approval

The University of Heidelberg received approval for the research from their human subjects committee in Germany (130/2002), which was extended in 2005 and 2008, as well as from the Nouna Health Research Center ethical committee (2005-005/CLE/CRSN).

## Results

### Enrolment into CBHI

The analysis was based on 33,500 children who fulfilled the criteria of being born in 2000 or later and having spent time in the HDSS study area between their second month and fifth year of life. The proportion of children in the present sample who had ever been enrolled in CBHI during the observation period was about 4.8%. About 74% of those children were insured until the end of the observation period and 95% of the insured children were continuously insured without disruption. Table 1 shows the distribution of sample characteristics and the factors that affected enrolment in health insurance. The strongest factors determining CBHI enrolment in the present analysis were SES, educational level of the father, distance to the reference health facility, year of birth, and insurance status of the mother at the time of birth. Mother's enrolment in the plan at the time of the child's birth was by far the strongest predictor, where children of enrolled mothers were 14 times more likely to be enrolled than children of non-enrolled mothers. A trend in enrolment was found for SES, where the probability of enrolment decreased consistently by quintile. Children in the poorest quintile were least likely to enrol (HR=0.40) as compared to the least poor quintile.^1^ As expected, year of birth had a strong effect on enrolment (HR=1.76 per 5 years), partly explained by the fact that insurance was only available to all HDSS residents from 2006 onwards. About 30% of the sample lived in the town of Nouna, where enrolment probability was 60% higher than in the villages. This effect was partly explained by the different educational attainment of the parents, leading to a decreased HR of 1.25 after adjustment. Likewise, the effect of mother's education, age of father at birth, and the number of siblings was strongly attenuated by adjustment for other variables. There was no association between enrolment and sex, season of birth, twin birth, household size, age of mother at birth, or previous death of mother.

**Table 1:** Factors affecting enrolment into health insurance in 33,500 children between 1 month and 5 years of age

			Crude analysis		Adjusted analysis	
			
Variable	Category	*N*	Hazard ratio	*p*	Hazard ratio	*p*
Residence	Rural	23,691	1		1	
	Nouna (town)	9,809	1.60	<0.0001	1.25	0.0007
Ethnicity	Dafing	12,539	1		1	
	Bwaba	7,262	0.82	0.0026	0.85	0.073
	Mossi	6,675	0.61	<0.0001	0.58	<0.0001
	Peulh	3,350	0.93	0.37	0.89	0.17
	Samo	2,687	0.87	0.16	0.69	0.0002
	Other	987	1.40	0.011	1.00	0.97
Religion	Muslim	22,157	1		1	
	Catholic	8,448	1.03	0.59	1.07	0.39
	Protestant	1,190	1.18	0.19	1.31	0.053
	Animist/Other	1,705	0.51	<0.0001	0.69	0.029
Distance	≤5 km	20,362^a^	1		1	
	>5 km	13,138^a^	0.39	<0.0001	0.57	<0.0001
Socioeconomic status	Q5 (least poor)	6,897	1		1	
	Q4	6,048	0.73	<0.0001	0.73	<0.0001
	Q3	4,831	0.74	<0.0001	0.69	<0.0001
	Q2	4,183	0.49	<0.0001	0.49	<0.0001
	Q1 (poorest)	3,611	0.41	<0.0001	0.40	<0.0001
	Missing	7,930	0.40	<0.0001	0.43	<0.0001
Education of mother	None	20,637	1		1	
	Basic	3,007	1.72	<0.0001	1.19	0.024
	Secondary	930	2.40	<0.0001	1.16	0.26
	Missing	8,926	0.88	0.59	1.07	0.48
Education of father	None	18,003	1		1	
	Basic	4,746	2.03	<0.0001	1.69	<0.0001
	Secondary	1,183	4.04	<0.0001	1.87	<0.0001
	Missing	9,568	0.99	0.91	1.00	0.99
Age of father	20–40	21,460	1		1	
	<20	535	0.83	0.37	1.03	0.90
	41+	6,791	0.83	0.0036	0.92	0.26
	Missing	4,714	0.84	0.039	0.93	0.58
Birth year	Before 2006	18,690	1.18		1.12	
	2006 and after	14,810	(per year)	<0.0001	(per year)	<0.0001
Mother insured at birth	Not insured	33,029	1		1	
	Insured	471	23.65	<0.0001	13.81	<0.0001
Number of older siblings	1–3	14,592	1		1	
	None	8,849	0.90	0.077	0.94	0.33
	4 or more	7,734	0.77	<0.0001	0.88	0.058
	Missing	2,325	0.90	0.33	1.06	0.72
Spacing to next younger sibling	18+ months or none	32,478	1		1	
	<18 months	1,022	0.78	0.15	0.69	0.032

The following variables showed no effects: sex, season of birth, twin birth, household size, age of mother, or previous death of mother.

a For frequency measures, distance measured in 2005.

### Mortality

**Fig. 2:**
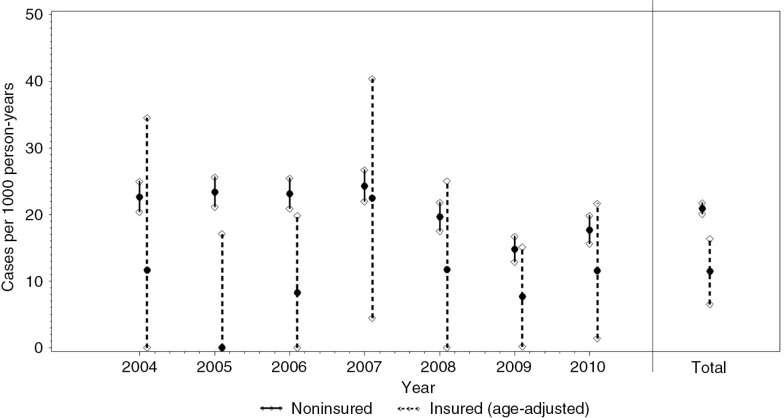
Mortality rates between 2004 and 2010 among enrolled (age-adjusted rates) and non-enrolled children, based on 2,285 enrolled and 113,041 non-enrolled person-years.

Average child mortality during the first 5 years of life in the non-enrolled children was about 21 deaths per 1,000 person-years (Fig. 2). Age-standardised mortality rate during the same period among children enrolled in CBHI was significantly lower, with about 11 deaths per 1,000 person-years. Due to the low number of enrolled children, the rates were inconsistent over the years and confidence intervals were wide.

In the Cox proportional hazards regression, the risk of death before the age of 5 years was significantly lower in insured children as compared to their non-insured counterparts (HR=0.45). This difference changed to a value of 0.54 (95% CI 0.43–0.68) after adjustment for possible confounders (Table 2). The most relevant confounders we identified were year of birth, distance to the reference health facility, and educational attainment of the father. However, adjustment for these factors did not strongly attenuate the results.

**Table 2:** The effect of insurance and insurance-related factors on under-five mortality in 33,500 children between 1 month and 5 years of age

			Crude analysis		Adjusted analysis	
			
Variable	Category	*N*	Hazard ratio	*p*	Hazard ratio	*p*
Insurance	Never insured	31,899	1		1	
	Insured at some point	1,601	0.45	0.0002	0.54	0.0066

Adjusted for rural-urban residence, ethnicity, religion, distance to the reference health facility, socioeconomic status, educational attainment of mother and father, age of father at time of birth, year of birth, community-based health insurance enrolment of the mother at time of birth, number of older siblings, and birth spacing to next younger sibling.

Stratified analyses were performed for those factors most strongly associated with enrolment and mortality. HRs mainly remained unchanged in the different stratification groups (Supplementary Table 1). Larger deviations from the original value of 0.54 were not significant and could be explained by low sample size.

The sensitivity analysis in 6,494 children with known vaccination status at age 1 revealed a strong association between vaccination coverage and insurance enrolment as well as child mortality. However, the impact of insurance enrolment on mortality was not affected by adjustment for vaccination coverage.

## Discussion

This study analysed the impact of CBHI enrolment on childhood mortality in sub-Saharan Africa. Remarkably, children enrolled in CBHI had a mortality rate almost 50% less than their non-enrolled counterparts.

There is very little published data on the association between health insurance enrolment and child health, especially from developing countries. The present analysis found the strongest effect shown to date of health insurance enrolment on mortality. A small number of other studies have analysed the effect of health insurance enrolment on measures of child health.

A previously published study from the Nouna HDSS analysed the effect of CBHI on mortality, using insurance availability at the village level as an explanatory variable and child mortality at the village level as an outcome variable. The authors found only a small and non-significant effect of insurance availability on child mortality. This finding was likely a result of the small sample size and low number of enrolled individuals in the clusters where insurance was available (14). Likewise, a different ecological study from Costa Rica on the same subject found no significant effect, whereas one study from Rwanda showed a small reduction of 3–14% in the mortality of enrolled infants (18, 19). Two studies from Vietnam and the Philippines found significant protective effects of health insurance enrolment on weight for age (20) and wasting and infections after hospital discharge (21).

There are in principle two major theories that could explain the effect of health insurance enrolment on mortality: 1) increased utilisation of health services and 2) reduction of out-of-pocket expenditure on health services and medication, opening the opportunity to spend this money for other health-related goods such as food, housing, or clothes (20). There is general agreement on the first theory, and evidence from Burkina Faso shows a 40% increase in the utilisation of outpatient services among enrolled individuals (7, 8, 22). A recent study from the Nouna area in Burkina Faso also provides evidence for the second theory, where insurance enrolment was shown to decrease annual health expenditure by $3.80 on average (14). This finding is in line with other research on health expenditure, which generally finds protective effects for health insurance enrolment (7, 22).

Malaria is the main cause of mortality in the Nouna study area, with more than 40% of all childhood deaths being attributed to this disease (23). There have been major improvements in the rollout of malaria prevention interventions in recent years, in particular regarding coverage with insecticide-treated bed nets, but the use of early diagnosis and effective treatment with artemisinin-based combination therapy (ACT) has remained rather low (24, 25). Because the high childhood mortality due to malaria in rural sub-Saharan Africa is mainly caused by late diagnosis and treatment as well as by treatment with ineffective drugs, the availability of free malaria diagnosis and ACT treatment in the frame of the CBHI pilot program in Nouna could well have played a major role regarding the significantly lower mortality we observed in insured compared to uninsured children (26). This assumption is supported by limited information on the cause of death, which was available for about 77% of the deceased children from a routinely applied verbal autopsy questionnaire (10). In the group of non-enrolled children, about 60% of all deaths were attributed to malaria, compared to only 41% of all deaths in enrolled children.

The present analysis was based on observational data, where individuals self-selected insurance enrolment, which could have biased the results (27). It has been shown that adverse selection plays a major role in insurance enrolment, as in some settings sick and especially chronically ill individuals are more likely to enrol into insurance schemes (adverse selection) (28). This type of bias cannot, however, explain the strong protective effect of insurance enrolment found in the present analysis, as this bias would at most have attenuated the effect.

Of higher concern is a possible self-selection of individuals with lower mortality risk into the CBHI scheme. As in a previous study from Nouna HDSS, it could be shown in the present analysis that indeed more highly educated persons with higher SES, and living closer to the reference health facility decided to enrol in CBHI (Table 1) (12). These factors have also been shown to be associated with lower child mortality in the present study area as well as in similar settings (29, 30). However, adjustment only slightly attenuated the association between insurance enrolment and child mortality, and there were no significant differences in the stratified analyses, indicating an independent association between insurance enrolment status and child mortality. For an additional sensitivity analysis, we calculated propensity scores based on the first model and performed the main analysis stratified by quintiles of this propensity score. This method yielded identical results to the original analysis.

Further limitations of the present study come from the facts that 1) only 4.8% of the children had been insured for some period and 2) not all children enrolled in insurance could be linked with the routine HDSS database. To address the first limitation, we investigated possible structural differences between insured and non-insured. This analysis is naturally restricted by the variables available for analysis. We consider it unlikely that the mortality difference can be explained by other, non-observed factors, but we also cannot fully exclude this. Regarding the second limitation, with a successful matching proportion of 89%, this means that about 11% (*N=*241) of the enrolled children were erroneously classified as non-enrolled. It does not seem likely that mortality itself was related to matching success between the databases. Non-differential misclassification is generally associated with an underestimation of effects and would rather have attenuated the real effect of enrolment on mortality.

Several factors could not be considered in the present analysis, such as cultural beliefs, illness perception, use of traditional medicine, and previous experiences with health facilities. These factors could be associated with care-seeking behaviour and could therefore have influenced both insurance enrolment and child mortality (31). It was shown that vaccination coverage, which was used as a proxy for health behaviour, was strongly associated with insurance enrolment as well as child mortality. However, the effect of insurance enrolment on child mortality was not changed by adjustment for vaccination coverage. Previous positive or negative experience with health facilities would probably be most strongly associated with the reference health facility itself. However, a sensitivity analysis with adjustment for the reference health facility did not influence the presented estimates.

This is the first study showing a strong effect of health insurance enrolment on the survival of young children in a developing country. For a deeper insight into the reasons, further studies should incorporate health service utilisation and household expenditure on different health-related and other goods into their analyses. If the association shown here is real and can be extrapolated to similar settings, strong efforts should be undertaken to maintain existing insurance schemes and make enrolment affordable for the whole population. Given the magnitude of the effect shown, this strategy could be a major driving factor in the reduction in child mortality in the developing world, especially in countries like Burkina Faso that have had difficulties staying on track to achieve Millennium Development Goal 4, the reduction of child mortality by two-thirds between 1990 and 2015.

## Supplementary Material

Health insurance and child mortality in rural Burkina Faso

## Footnotes

1 HR, hazard ratio
