# Supplementary material for: Health insurance and child mortality in rural Burkina Faso
Source: Glob Health Action. 2015 Apr 28;8:10.3402/gha.v8.27327. doi: 10.3402/gha.v8.27327 (PMC4414785; doi:10.3402/gha.v8.27327)
Supplement: Health insurance and child mortality in rural Burkina Faso [file GHA-8-27327-s001.pdf]

Supplementary Table 1: Effect of health insurance enrolment on child mortality in strata of the variables, most strongly associated with CBHI enrolment in 33 500 children from the Nouna HDSS

Stratification Variable	*Category*	*N*	*Hazard Ratio*	*P-Value*
Distance	≤5km	20 362*	0.56	0.019
	>5km	13 138*	0.47	0.20
Residence	Rural	23 691	0.57	0.025
	Nouna town	9809	0.51	0.18
SES	Q5 (least poor)	6897	0.59	0.15
	Q4	6048	0.94	0.90
	Q3	4831	0.28	0.074
	Q2	4183	0.83	0.88
	Q1 (poorest)	3611	0.32	0.27
Education of father	None	18 003	0.51	0.039
	Basic	4746	0.58	0.29
	Secondary	1183	0.52	0.53
Birthyear	2000	3092	0.00	0.96
	2001	3037	0.00	0.97
	2002	3024	0.00	0.97
	2003	3216	0.40	0.19
	2004	3134	0.00	0.96
	2005	3187	1.05	0.91
	2006	3096	0.56	0.27
	2007	3311	0.80	0.64
	2008	3111	0.00	0.97
	2009	2688	1.61	0.40
	2010	2604	0.00	0.996
Mother insured at birth	Non-insured	33 029	0.51	0.010
	Insured	471	0.66	0.43

*For frequency measures: distance in year 2005
